# Effets du Suivi des Enfants « Hors Zone » Vaccinés sur la Qualité des Données, District Sanitaire de San Pedro, Côte D'ivoire, 2019

**DOI:** 10.48327/mtsibulletin.2021.103

**Published:** 2021-05-19

**Authors:** A. Aplogan, D. Palenfo, J. Koala, N. Gouda, A. Essoh, R. Touré, K.D. Ekra

**Affiliations:** 1 Agence de médecine préventive Afrique, 08 BP 660 Abidjan 08, Côte d'Ivoire; 2 Projet HIGHS CDC, US Embassy Abidjan, BP 730 Abidjan Cedex 03, Côte d'Ivoire; 3 Direction de la coordination du PEV, 22 BP 458 Abidjan, Côte d'Ivoire

**Keywords:** Effets, Suivi, Enfants « hors zone » vaccinés, Qualité des données, San Pedro, Côte d'Ivoire, Afrique subsaharienne, Effects, Monitoring, ‘'out-of-area'' vaccinated children, Data quality, San Pedro, Côte d'Ivoire, Sub-Saharan Africa

## Abstract

En vue d'améliorer la qualité des données de vaccination des centres de santé, nous avons réalisé le suivi des enfants « hors zone » vaccinés dans le district sanitaire de San Pedro. L'objectif de ce travail était de mesurer les effets de la prise en compte des enfants « hors zone » vaccinés sur la qualité des données et les performances vaccinales des centres de santé. Ce suivi qui avait été fait entre mars et août 2019, avait comporté quatre étapes: la vaccination des enfants « hors zone » par le centre de santé, la mise à jour mensuelle dans le registre du statut des enfants « hors zone » vaccinés, la comptabilisation de ces enfants et la réestimation des performances vaccinales du centre de santé. Au total 37 des 40 centres du district avaient eu 980 enfants « hors zone » vaccinés soit 5,7% de la cible vaccinale. Le quart de ces enfants résidait en dehors du district. La vaccination des enfants « hors zone » avait concerné tous les vaccins du PEV mais surtout le BCG, le DTC-HepB-Hib et le pneumocoque_13. Les effectifs des enfants « hors zone extra district » vaccinés n'avaient pas modifié les couvertures vaccinales du district. Par contre, dans les centres de santé concernés, la précision des données avait été améliorée dans 65% des centres pour le DTC-HepB-Hib_1, 70% des centres pour le RR et 62% des centres pour le taux d'abandon global de vaccination. L'approche utilisée avait permis d'améliorer la qualité des données de vaccination des centres de santé, sans coût additionnel.

## Abstract (en)

In order to improve the quality of vaccination data from the health centers, we carried out the follow-up of “out-of-area” children, vaccinated in the district of San Pedro. The objective of this work was to measure the effects of taking into account the “out-of-area” vaccinated children on the quality of the data and the immunization performance of the health centers. This monitoring, which was carried out between March and August 2019, consisted of four steps: the vaccination of “out-of-area” children by the health center, the monthly update in the register of the status of vaccinated “out-of-area” children, the accounting of these children, and the re-evaluation of the health center's vaccine performance. A total of 37 of the 40 district centers had 980 “out-of-area” vaccinated children, representing 5.7% of the immunization target. The quarter of these children resided outside the district. The vaccination of “out-of-area” children included all EPI vaccines, but especially BCG, DTP-HepB-Hib and Pneumococcus_13. The number of “outside the extra-district area” vaccinated children had not changed the district's immunization coverage. On the other hand, in the health centers concerned, the accuracy of the data had been improved in 65% of the centers for DTP-HepB-Hib_1, in 70% of the centers for RR and in 65% of the centers for the overall immunization dropout rate. The approach used had made it possible to improve the quality of immunization data from health centers at no additional cost.

## Introduction

Selon l'Organisation mondiale de la santé, les avantages de la vaccination doivent s'appliquer à tous de manière équitable [6], d'où la nécessité de s'associer aux communautés pour fournir les services de vaccination les mieux adaptés aux besoins locaux [5] et d'investir dans des stratégies idoines pour identifier les enfants incomplètement vaccinés [4].

Le suivi des enfants « hors zone » vaccinés et la qualité des données sont des problèmes majeurs pour les programmes élargis de vaccination (PEV) en Afrique. En Côte d'Ivoire par exemple, certains districts de santé présentent: des couvertures vaccinales supérieures à 100%; des taux d'abandon de vaccination élevés ou négatifs; des discordances entre les données administratives de couverture vaccinale et les résultats d'enquête. Malgré l'importance de ces problèmes, il existe peu de publications sur les approches utilisées pour les résoudre.

Les supervisions réalisées dans ces districts ont permis de comprendre que ces constats sont dus à des problèmes: de qualité de remplissage des outils de recueil et de rapportage des données; d'estimation des populations cibles; de mobilité de la population.

En Côte d'ivoire, la mobilité de la population est liée entre autres: à la quête du centre de santé le plus proche; au planning des séances de vaccination qui ne convient pas; à la qualité du service de vaccination (accueil, attente, attitude de l'agent); aux événements socio-culturels (baptême, mariage, funérailles, fête religieuse). Cette mobilité difficile à contrôler complique le suivi des enfants vaccinés et réduit la qualité des indicateurs de performance vaccinale des structures de santé concernées.

**Figure 1:**
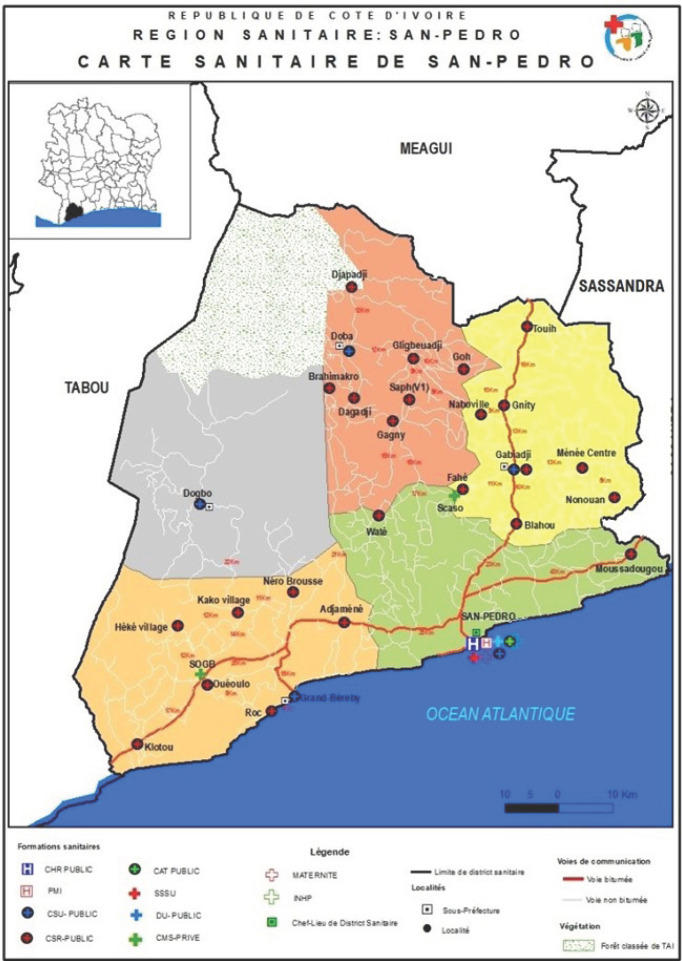
Carte du district sanitaire de San Pedro Map of the San Pedro Health District

C'est pour améliorer la qualité des données de vaccination qu'une approche de suivi des enfants « hors zone » vaccinés avait été conçue et testée dans le district sanitaire de San Pedro (Fig. 1). Il s'était agi de mesurer dans ce district les effets de la prise en compte des enfants « hors zone » vaccinés sur la qualité des données et les performances vaccinales des centres de santé.

## Méthodologie

Nous avons considéré comme enfants « hors zone », des enfants qui se présentent dans un centre de santé auquel leur zone de résidence n'est pas administrativement rattachée. Les enfants « hors zone » résidant dans le district sont les enfants « hors zone intra district » et ceux qui résident en dehors du district sont les enfants « hors zone extra district ».

Les centres de santé concernés par le phénomène d'enfants « hors zone » vaccinés sont ceux qui ont vacciné des enfants provenant d'une autre aire et/ou ceux dont des enfants ont été vaccinés par un autre centre de santé.

Le suivi des enfants « hors zone » vaccinés a été mis en oeuvre entre mars et août 2019 dans le district sanitaire (DS) de San Pedro qui est situé à l'extrême sud-ouest de la Côte d'Ivoire, à 334 kilomètres d'Abidjan et a une superficie de 6720 km^2^. En 2018, il avait une population de 753027 habitants dont 43% d'étrangers, 27203 enfants de 0 à 11 mois et un taux de non migrant de 37,1%. Il disposait d'un centre hospitalier régional, de quarante centres de santé, d'une maternité et de vingt-six pharmacies. San Pédro est le deuxième pôle économique de la Côte d'Ivoire après Abidjan, en raison de son port, de la présence d'usines cacaoyère, de minoterie, de ciment, de bois et du tourisme

L'approche utilisée a comporté quatre étapes [2]: l'établissement par le centre de santé de la liste des enfants « hors zone » vaccinés selon la provenance; la mise à jour mensuelle du registre de vaccination du centre la comptabilisation des enfants « hors zone » vaccinés du centre et la réestimation des performances du centre.

**Figure 2:**
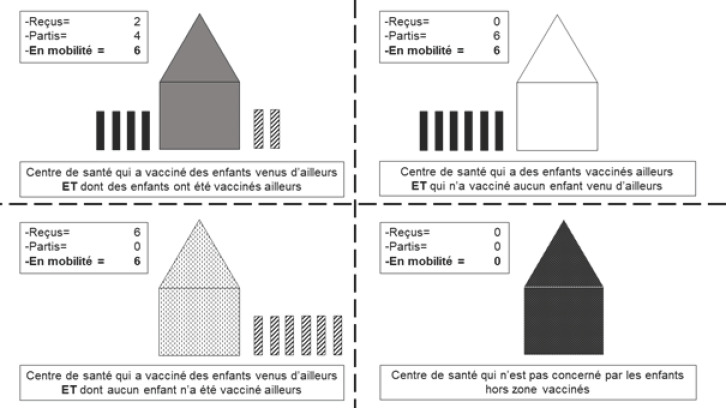
Catégories de centres de santé par rapport aux enfants « hors zone » vaccinés Categories of health center in relation to « out-of-area » vaccinated children

Nous avons classé les centres de santé en quatre catégories: les centres qui ont vacciné des enfants venus d'ailleurs et dont des enfants ont été vaccinés ailleurs; les centres qui ont des enfants vaccinés ailleurs et qui n'ont vacciné aucun enfant venu d'ailleurs; les centres qui ont vacciné des enfants venus d'ailleurs et dont aucun enfant n'a été vacciné ailleurs; les centres qui ne sont pas concernés par les enfants « hors zone » vaccinés (Fig. 2).

Nous avons calculé les proportions d'enfants « hors zone » vaccinés intra et extra district, selon la classification des centres de santé, par rapport aux vaccins reçus. Nous avons aussi calculé les proportions de centres de santé selon l'évolution de leurs performances après la prise en compte des enfants « hors zone » vaccinés.

Pour apprécier l'évolution de la performance des centres après la prise en compte des enfants « hors zone » vaccinés nous avons retenu trois indicateurs: la couverture DTC-HepB-Hib_1 qui évalue l'accès au PEV, la couverture rougeole-rubéole qui évalue la capacité du PEV à atteindre les enfants de moins d'un an et le taux d'abandon global de vaccination qui évalue la continuité d'utilisation des services du PEV.

## Résultats

### Répartition des enfants « hors zone » vaccinés selon leur résidence dans le district ou en dehors

De mars à août 2019, 37 des 40 centres de santé du district de San Pedro, étaient concernés par le phénomène d'enfants « hors zone » vaccinés.

Les enfants « hors zone » vaccinés étaient au nombre de 980 soit 5,7% de la cible vaccinale des 40 centres (n= 17398) et 8,2% de la cible vaccinale des 37 centres (n= 12070).

Sur ces 980 enfants « hors zone » vaccinés, 240 (25%) résidaient hors du district et avaient été vaccinés dans les centres de santé du district de San Pedro. Ces 240 enfants représentaient 1,3% de la cible vaccinale du district de San Pedro.

### Répartition des centres de santé et des enfants « hors zone » vaccinés par catégorie de centre

Durant cette période: 27 centres de santé (68%) avaient vacciné des enfants provenant d'une autre aire et avaient eu des enfants vaccinés par un autre centre; 7 centres (18%) avaient eu des enfants vaccinés par un autre centre alors qu'ils n'avaient vacciné aucun enfant provenant d'une autre aire; 3 centres (7%) avaient vacciné des enfants provenant d'une autre aire alors qu'aucun de leurs enfants n'avait été vacciné par un autre centre; 3 centres (7%) n'avaient vacciné aucun enfant provenant d'une autre aire et dont aucun enfant n'avait été vacciné par un autre centre (Tableau I).

**Tableau I:** Répartition des centres de santé et des enfants « hors zone » vaccinés par catégorie de centre, District de San Pedro, mars à août 2019 (n= 40) Distribution of health centers and “out-of-area” vaccinated children by center category, San Pedro District, March to August 2019 (n= 40)

	Nb de centres	Cible vaccinale	Enfants « hors zone » vaccinés	
			nombre	%
**Centres ayant vacciné des enfants provenant d'une autre aire et dont des enfants ont été vaccinés par un autre centre**	27	4621	881	19%
**Centres dont des enfants sont vaccinés par un autre centre et qui n'ont vacciné aucun enfant provenant d'une autre aire**	7	3882	412	11%
**Centres ayant vacciné des enfants provenant d'une autre aire et dont aucun enfant n'a été vacciné par un autre centre**	3	3567	427	12%
**Centre n'ayant vacciné aucun enfant provenant d'une autre aire et dont aucun enfant n'a été vacciné par un autre centre**	3	5328	0	0%

### Répartition des enfants « hors zone » vaccinés selon les vaccins reçus

Les enfants « hors zone » vaccinés avaient reçu tous les types de vaccin (Tableau II).

**Tableau II:** Répartition des enfants « hors zone » vaccinés selon les vaccins reçus, District de San Pedro, mars-août 2019 Distribution of “out-of-area” children vaccinated according to vaccines received, San Pedro District, March-August 2019

	Proportion d'enfants « hors zone intra district » vaccinés (n= 740)				Proportion d'enfants « hors zone extra district » vaccinés (n= 240)			
Vaccins	dose 0	dose 1	dose 2	dose 3	dose 0	dose 1	dose 2	dose 3
**BCG**	-	18%	-	-	-	23%	-	-
**VPO**	16%	9%	6%	3%	9%	15%	13%	15%
**DTC-HepB-Hib**	-	12%	8%	4%	-	31%	28%	23%
**PCV13**	-	10%	7%	6%	-	17%	16%	8%
**Rotarix**	-	9%	6%	-	-	16%	18%	-
**VPI**	-	2%	-	-	-	8%	-	-
**RR, VAA, Men A**	-	2%	-	-	-	29%	-	-

Quel que soit le type de vaccin, la proportion d'enfants qui les avait reçus était plus élevée chez les « hors zone extra district » que chez les « hors zone intra district ».

Les vaccins reçus par au moins 10% des enfants « hors zone intra district » étaient; le BCG (18%), le VPO_0 (16%), le DTC-HepB-Hib_1 (12%) et le pneumocoque13_1 (10%). Quant aux enfants « hors zone extra district », les vaccins reçus par au moins 20% d'entre eux étaient: le DTC-HepB-Hib_1 (31%), le RR, VAA et Men A (29%), le DTC-HepB-Hib_2 (28%), le DTC-HepB-Hib_3, et BCG (23%).

### Performances vaccinales du district après la prise en compte des enfants « hors zone » vaccinés

La prise en compte des enfants « hors zone extra district » vaccinés n'a eu aucun effet sur les couvertures vaccinales du district de San Pedro. En effet, en retirant des nombres d'enfants vaccinés dans le district ceux qui y étaient vaccinés mais qui résidaient dans d'autres districts, les couvertures vaccinales du district étaient inchangées (Tableau III).

**Tableau III:** Performances vaccinales du district de San Pedro avec et sans les enfants « hors zone » vaccinés qui résidaient dans d'autres districts, mars-août 2019 (n= 17398) Vaccine performance in San Pedro district with and without vaccinated “out-of-area” children who resided in other districts, March-August 2019 (n= 17,398)

Couvertures vaccinales des enfants du district		
	Avec les « hors zone extra district »	Sans les « hors zone extra district »
**BCG**	84%	84%
**VPO_0**	85%	84%
**DTC-HepB-Hib_1**	82%	83%
**DTC-HepB-Hib_3**	67%	67%
**Rotarix_1**	78%	78%
**Rotarix_2**	71%	71%
**Rougeole-rubéole**	72%	72%
**VAA**	69%	68%
**MenA**	65%	65%

En revanche, la prise en compte des enfants « hors zone » vaccinés avait permis de rendre plus exacte la couverture vaccinale DTC-HepB-Hib_1 dans 24 centres (65%) dont 10 centres dans lesquels elle avait augmenté et 14 où elle avait diminué. Pour la couverture rougeole-rubéole, elle était devenue plus exacte dans 26 centres (70%) dont 5 centres dans lesquels elle avait augmenté et 21 où elle avait diminué. Quant au taux d'abandon global de vaccination, il était devenu plus exact dans 23 centres (62%) dont 10 centres dans lesquels il avait augmenté et 13 où il avait diminué (Tableau IV).

**Tableau IV:** Répartition des centres de santé selon l'évolution de leurs performances après la prise en compte des enfants « hors zone » vaccinés, District de San Pedro, mars-août 2019 (n = 37) Distribution of health centers according to the evolution of their performance after taking into account “out-of-area” vaccinated children, San Pedro District, March-August 2019 (n = 37)

	Couverture vaccinale DTC-HepB-Hib_1	Couverture vaccinale rougeole-rubéole	Taux d'abandon global de vaccination
**Centres avec une performance améliorée**	10 (27%)	5 (13%)	10 (27%)
**Centres avec une performance en baisse**	14 (38%)	21 (57%)	13 (35%)
**Centres avec une performance inchangée**	13 (35%)	11 (30%)	14 (38%)

## Discussion

Dans le district de San Pedro, entre mars et août 2019, le phénomène des enfants « hors zone » vacciné était important puisqu'il avait concerné 93% des centres de santé et 5,7% de la cible vaccinale. Ceci pourrait s'expliquer par le fait que durant cette période qui correspond à la saison des pluies, les populations migraient dans les zones d'activités agricoles, s'y installaient pour des durées variables et y faisait donc vacciner leurs enfants.

Les ¾ des enfants « hors zone » vaccinés résidaient à San Pedro alors que le ¼ restant provenait d'autres districts. Le phénomène des enfants « hors zone » vaccinés, n'était pas spécifique à certains vaccins ou à certaines doses de vaccin. Il avait concerné tous les âges et toutes les doses de tous les vaccins.

Du fait de ce phénomène, certains centres présentaient des couvertures vaccinales élevées qui en réalité étaient faussées par un apport d'enfants « hors zone » vaccinés. Les agents de santé de ces centres pensaient à tort qu'ils avaient vacciné toute leur cible et ne menaient donc pas d'actions spécifiques pour rechercher et vacciner les enfants « zéro dose » ou « perdus de vue ». Ceci avait pour conséquence l'accumulation de ces enfants non vaccinés ou partiellement vaccinés, ce qui augmente le risque de survenue d'épidémie des maladies à prévention vaccinale.

C'est pour cela qu'il est important que les agents de santé prennent en compte les enfants « hors zone » vaccinés, recherchent auprès de leurs pairs les informations sur ces enfants pour actualiser leur registre de vaccination et réestimer les performances de leurs centres. Cela leur permettra le cas échéant, de prendre conscience de l'existence de poches d'enfants « zéro dose » ou « perdus de vue » et de focaliser leurs interventions sur ces poches. Ces interventions pourraient s'appuyer sur l'utilisation des agents de santé communautaire ou des membres des organisations de la société civile comme ce fut le cas dans l'étude de Aplogan et al [1]. Par ailleurs, dans leur publication, Atkinson et Cheyne ont montré que le recours aux agents de santé communautaire, avait été couronné de succès pour régler les problèmes d'accès à la vaccination en milieu urbain [3].

Le constat selon lequel quel que soit le type de vaccin, la proportion d'enfants qui les avait reçus était plus élevée chez les « hors zone extra district » que chez les « hors zone intra district » méritent des études complémentaires pour l'expliquer.

La prise en compte des enfants « hors zone » vaccinés résidant en dehors du district de San Pedro, n'avait pas modifié les couvertures vaccinales de ce dernier. Ceci pourrait être expliqué par le fait qu'à l'échelle du district les effectifs de ces enfants « hors zone extra district » vaccinés par antigène, étaient faibles par rapport à la cible vaccinale.

En revanche, dans plus de 60% des centres, la prise en compte des enfants « hors zone » vaccinés avait permis d'améliorer l'exactitude des couvertures vaccinales et des taux d'abandon de vaccination. Les responsables des centres dont les couvertures vaccinales étaient surestimées (38% pour DTC-HepB-Hib_1 et 57% pour rougeole-rubéole), devraient mettre en oeuvre des interventions adaptées pour retrouver leurs cibles manquantes.

## Conclusion

L'approche de suivi des enfants « hors zone » vaccinés, mis en place dans le district de San Pedro en Côte d'Ivoire a permis d'améliorer la précision des données de vaccination des centres de santé et à leurs responsables d'adapter leurs interventions aux performances réelles des centres. Par ailleurs, la mise en oeuvre de cette approche n'a engendré aucun coût additionnel.

## Conflits D'intérêts

Les auteurs ne déclarent aucun conflit d'intérêt.
